# No yield penalty under favorable conditions paving the way for successful adoption of flood tolerant rice

**DOI:** 10.1038/s41598-018-27648-y

**Published:** 2018-06-18

**Authors:** Manzoor H. Dar, Najam W. Zaidi, Showkat A. Waza, Satish B. Verulkar, T. Ahmed, P. K. Singh, S. K. Bardhan Roy, Bedanand Chaudhary, Rambaran Yadav, Mirza Mofazzal Islam, Khandakar M. Iftekharuddaula, J. K. Roy, R. M. Kathiresan, B. N. Singh, Uma S. Singh, Abdelbagi M. Ismail

**Affiliations:** 1 International Rice Research Institute (IRRI-India), NASC Complex, New Delhi, India; 2 Sher-e-Kashmir University of Agricultural Sciences & Technology of Kashmir (J&K), Kashmir, India; 3 Indira Gandhi Krishi Vishwavidyalaya, Raipur, Chhattisgarh India; 4 Assam Agricultural University, Jorhat, Assam India; 5 Banaras Hindu University, Varanasi, Uttar Pradesh India; 6 Center for Strategic Studies, Kolkata, West Bengal India; 7 Regional Agricultural Research Station, NARC, Tarahara, Nepal; 8 Regional Agriculture Research Station, NARC, Bara, Nepal; 9 Bangladesh Institute of Nuclear Agriculture, Mymensingh, Bangladesh; 10 Bangladesh Rice Research Institute, Gazipur, Bangladesh; 11 Association for Integrated Development (AID), Bhubaneswar, Odisha India; 12 Annamalai University, Tamil Nadu, India; 13 Centre for Research and Development (CRD), Gorakhpur, UP India; 14 International Rice Research Institute (IRRI), Los Banos, Philippines

## Abstract

Flooding is one of the major constraints for rice production in rainfed lowlands, especially in years and areas of high rainfall. Incorporating the Sub1 (Submergence1) gene into high yielding popular varieties has proven to be the most feasible approach to sustain rice production in submergence-prone areas. Introgression of this QTL into popular varieties has resulted in considerable improvement in yield after flooding. However, its impact under non-flooded conditions or years have not been thoroughly evaluated which is important for the farmers to accept and adopt any new version of their popular varieties. The present study was carried out to evaluate the effect of Sub1 on grain yield of rice in different genetic backgrounds, under non-submergence conditions, over years and locations. The study was carried out using head to head trials in farmer’s fields, which enable the farmers to more accurately compare the performance of Sub1 varieties with their recurrent parents under own management. The data generated from different head to head trials revealed that the grain yield of Sub1 varieties was either statistically similar or higher than their non-Sub1 counterparts under non-submergence conditions. Thus, Sub1 rice varieties show no instance of yield penalty of the introgressed gene.

## Introduction

Present and future food demands necessitate a significant increase in the productivity of major food crops especially in the areas that are highly prone to environmental perturbations^1^. Among the major food crops, rice production is increasingly being affected by various environmental stresses, with over 30% of the 700 million poor households in rainfed lowlands being affected in Asia^2^. Flooding is one of the major constraints for rice production in the years and areas of high rainfall and at least 16% of the world’s rice production area is affected by frequent submergence due to flash floods^3,4^. Moreover, the long-term adverse effects of climate change are anticipated to aggravate flooding incidences, a major challenge for sustainable rice production in future^5^. Many areas are expected to witness an increase in flooding as a consequence of sea level rise, uneven distribution of rainfall and predicted increases in frequencies and intensities of extreme weather events. The overall consequences will be more serious in the rainfed tropics, where poverty and food insecurity are very common^6,7^.

Although, rice is well known for its ability to grow in flooded soils, most rice cultivars cannot survive submergence for more than a week^8^. Flash floods leading to complete submergence of the plants for 10–15 days is one of the major recurring problems for rice production in rainfed lowlands of south and south-east Asia^9^. Among the various rainfed rice-growing ecologies, India, Nepal and Bangladesh occupy one of the largest submergence prone areas in Asia^10^. The farmers in these areas cultivate popular rice varieties such as Swarna, Samba Mahsuri, IR64 and MTU 1010 for their high yield, desirable grain quality and better market value. However, these varieties are highly sensitive to submergence, leading to enormous losses every year due to recurrent floods^11^. To guard against these losses, some farmers still cultivate older and traditional varieties, which can withstand flooding to some extent but have lower yields and poorer quality. In some areas, farmers have abandoned rice cultivation altogether and preferred to leave their fields fallow during the wet season^12^. Incorporating submergence tolerance into high yielding popular varieties has proven to be the most effective approach to cope with and mitigate the effects of submergence.

Molecular mapping and quantitative trait locus (QTL) studies have revealed that Submergence1 (Sub1) locus is the major source of submergence tolerance in rice^13–15^. The submergence tolerant genotypes with introgressed Sub1 locus have three Ethylene Response Factor (ERF) genes, viz., Sub1A, Sub1B and Sub1C^1,15^. The main submergence inducible Sub1A gene associated with the tolerance is absent in all japonica and most indica rice accessions. The other two submergence inducible genes Sub1B and Sub1C are invariably present in all the rice accessions at Sub1 locus but are not associated with submergence tolerance. Thus, the intolerant genotypes possess Sub1B and Sub1C genes, but do not have the Sub1A gene^16–19^. The Sub1 and non-Sub1 genotypes differ in 182 kb interval on chromosome 9 possessing Sub1 locus^15^. Sub1A reduces ethylene production and gibberellic acid responsiveness, economizing carbohydrate reserves, thereby significantly sustains endurance during submergence^20,21^. Sub1A has two allelic forms in submergence tolerant and intolerant indica and aus accessions^15^. SubA1–1 is present only in submergence tolerant genotypes, whereas the Sub1A-2 allele is found in intolerant lines. Sub1A-1 and Sub1A-2 code for identical proteins, except for Ser186 in the submergence tolerant allele and Pro186 in the intolerant one. Sub1A-1 stimulates rapid, prolonged and prominent transcript accumulation in response to submergence, whereas Sub1A-2 provides a lower proportion of transcript generation due to the stress^15,20^.

Using marker assisted breeding (MAB), the International Rice Research Institute (IRRI) has successfully incorporated the Sub1 (Submergence1) QTL from an Indian landrace (FR13A) into the mega variety Swarna, which is commonly being grown in many parts of India^1,22,23^. The new version of Swarna, called Swarna-Sub1, can withstand complete submergence for over two weeks and recovers well once flood water recedes. After few years of evaluation in farmers’ fields, Swarna-Sub1 was commercially released in India in 2009. Simultaneously, Sub1 was incorporated into several other popular varieties grown in rainfed and flood-prone areas of South and South-East Asia. Sub1 versions of these popular rice varieties (e.g. Swarna-Sub1, Sambha Mahsuri-Sub1, BR11-Sub1, CR1009-Sub1) has been recognized to have contributing for stabilizing rice productivity in the rainfed lowlands of Eastern India, Nepal and Bangladesh^12,24,25^. These varieties can serve as the most promising technologies for alleviating poverty and food insecurity for communities living in flood-affected areas and are dependent on rice. This provides assurance that the problem of food insecurity can be addressed through the development and dissemination of these popular varieties after equipping them with submergence tolerance^23^.

Results from farmers’ field trials indicated that, on average, Swarna-Sub1 can produce more than 1.0 t ha^−1^ yield advantage in submergence-prone areas over the original Swarna. Under controlled conditions, a yield advantage of 1.65 t ha^−1^ was obtained from Swarna-Sub1 compared to Swarna^26^. In severe cases of flooding for more than 10 days, Swarna usually dies, whereas Swarna-Sub1 produces over 2 t ha^−1^ ^23^. Similar results have been recorded for the other popular varieties introgressed with the Sub1 QTL^27^. Apparently Sub1 versions of popular rice varieties undoubtedly perform better than their non-Sub1 counterparts under submergence. However, this performance under stress conditions should not be at the cost of yield under control or non-submergence conditions, since flooding usually occur once in 2–5 years. Previous studies have reported contrasting results showing favorable, unfavorable or even neutral effects of different genes incorporated in various crop species^28,29^. However, replete knowledge on the influence of Sub1 QTL on yield and yield related parameters in farmers’ fields not affected by floods is incomplete. For any major stress traits incorporated, we need to ascertain the yield gain or penalty under non-stress conditions before large-scale dissemination of new varieties carrying that particular trait. The present study was carried out to evaluate the effect of Sub1 QTL, incorporated in different genetic backgrounds, on grain yield of rice under non-submergence conditions, over years and locations. The study was carried out using head to head trials in farmers’ fields, which enables farmers to compare Sub1 varieties with their recurrent parents under own management systems.

## Materials and Methods

The present study was carried out over different locations in India, Nepal and Bangladesh during the wet seasons of 2014 and 2015. The experimental material consisted of the four popular varieties of rice— Swarna, Samba Mahsuri, CR1009 and BR11, along with their Sub1 versions. A head to head trial approach was used to evaluate performance of the Sub1 varieties in comparison with their non-Sub1 versions. The trials were conducted in farmers’ fields and the selection of farmers for conducting the trials was accomplished through different national partners (NARES, National Agricultural Research and Extension System) in their particular areas. In these head to head trials, the pair of varieties (near isogenic lines; NILs) to be compared were grown in the same field (half of the field for each variety) or in two adjoining fields with similar conditions. These trials were conducted intentionally in areas where floods are not so common to assess the potential positive or any negative impacts of incorporating Sub1 into these popular varieties. No major submergence stress was recorded in all trials during the two years of the study. Moreover, the trials were conducted in assured irrigated areas and thus no case of drought or water scarcity was observed.

For Swarna-Sub1 and Swarna, 32 head to head trials were conducted at different locations in India, Nepal and Bangladesh during 2014. In 2015, 11 head to head trials were conducted for this pair of NILs at different locations in India and Nepal (Table 1). For Samba Mahsuri-Sub1 and Samba Mahsuri, 15 trials were carried out during 2014 and 29 during 2015 (Table 2). These trials were also conducted at different locations in India and Nepal. Comparison of CR1009-Sub1 with CR1009 was carried out in India, with 5 trials during 2014 and 13 trials during 2015 (Table 3). During 2014, 18 head to head trials for BR11-Sub1 and BR11 were conducted at different locations in Bangladesh and 11 trials in 2015 in both India and Bangladesh (Table 4). All trials were conducted during the wet seasons.

**Table 1:** Comparisons of grain yield of Swarna-Sub1 and Swarna in head to head trials during the wet seasons of 2014 and 2015.

Location	2014					2015				
	No. of trials	Grain yield (t ha^−1^) of Swarna-Sub1		Grain yield (t ha^−1^) of Swarna		No. of trials	Grain yield (t ha^−1^) of Swarna-Sub1		Grain yield (t ha^−1^) of Swarna	
		Mean	SD	Mean	SD		Mean	SD	Mean	SD
Kolkata, West Bengal, India	3	7.1	0.31	6.9	0.21	—	—	—	—	—
Gorakhpur, Uttar Pradesh, India	3	5.0*	0.35	4.0	0.40	—	—	—	—	—
Titabar, Assam, India	5	5.6*	0.29	5.1	0.15	—	—	—	—	—
Dhamtari, Chhattisgarh, India	3	6.2*	0.42	5.2	0.25	—	—	—	—	—
Puri, Nimapara, Odisha, India	6	5.5	0.37	5.0	0.23	—	—	—	—	—
Varanasi, Uttar Pradesh, India	—	—	—	—	—	3	6.9	0.47	6.7	0.36
Tarhara, Sunsari, Nepal	3	6.3*	0.35	3.4	0.31	5	4.5*	0.40	3.4	0.30
Hardinath, Nepal	3	4.6*	0.42	3.8	0.15	3	4.7*	0.35	3.6	0.25
Torotpara, Gazipur, Bangladesh	3	3.9	0.40	4.2	0.23	—	—	—	—	—
Rangpur, Bangladesh	3	4.3	0.40	4.1	0.21	—	—	—	—	—

^*^Indicates that the grain yield is significantly higher than the parental variety at level, *p* = 0.05.

**Table 2:** Comparative yield of Samba Mahsuri-Sub1 and Samba Mahsuri under head to head trials during the wet seasons of 2014 and 2015.

Location	2014					2015				
	No. of trials	Grain yield (t ha^−1^) of Samba Mahsuri-Sub1		Grain yield (t ha^−1^) of Samba Mahsuri		No. of trials	Grain yield (t ha^−1^) of Samba Mahsuri-Sub1		Grain yield (t ha^−1^) of Samba Mahsuri	
		Mean	SD	Mean	SD		Mean	SD	Mean	SD
Kolkata, West Bengal, India	3	6.1	0.26	5.9	0.26	3	4.2	0.36	4.0	0.17
Gorakhpur, Uttar Pradesh, India	3	4.1	0.21	4.8	0.72	—	—	—	—	—
Puri, Odisha, India	3	6.4*	0.21	6.0	0.21	5	3.6	0.24	3.6	0.16
Varanasi, Uttar Pradesh, India	—	—	—	—	—	3	5.0	0.47	4.7	0.40
Annamalai, Tamil Nadu, India	—	—	—	—	—	10	3.9	0.49	3.8	0.48
Tarhara, Sunsari, Nepal	3	4.0	0.35	3.2	0.32	5	3.7	0.30	3.5	0.29
Hardinath, Nepal	3	4.5	0.40	4.2	0.15	3	4.5*	0.30	3.8	0.25

^*^Indicates that the grain yield is significantly higher than the parental variety at level, *p* = 0.05.

**Table 3:** Yield comparison of CR1009-Sub1 and CR1009 in head to head trials during the wet seasons of 2014 and 2015.

Location	2014					2015				
	No. of trials	Grain yield (t ha^−1^) of CR1009-Sub1		Grain yield (t ha^−1^) of CR1009		No. of trials	Grain yield (t ha^−1^) of CR1009-Sub1		Grain yield (t ha^−1^) of CR1009	
		Mean	SD	Mean	SD		Mean	SD	Mean	SD
Kolkata, West Bengal, India	—	—	—	—	—	3	4.8	0.35	4.5	0.31
Annamalai, Tamil Nadu, India	5	5.0*	0.34	4.3	0.34	10	5.9	0.45	5.6	0.40

*Indicates that the grain yield is significantly higher than the parental variety at level, *p* = 0.05.

**Table 4:** Comparisons of grain yield of BRRI Dhan-52 (BR11- Sub1) and BR11 under head to head trials during the wet seasons of 2014 and 2015.

Location	2014					2015				
	No. of trials	Grain yield (t ha^−1^) of BRRI Dhan-52		Grain yield (t ha^−1^) of BR11		No. of trials	Grain yield (t ha^−1^) of BRRI Dhan-52		Grain yield (t ha^−1^) of BR11	
		Mean	SD	Mean	SD		Mean	SD	Mean	SD
Titabar, Assam, India	—	—	—	—	—	8	5.7	0.27	5.5	0.30
Torotpara, Gazipur, Bangladesh	3	4.0	0.15	4.1	0.26	3	4.8	0.25	5.0	0.35
Valuka, Gazipur, Bangladesh	3	5.1	0.32	4.9	0.26	—	—	—	—	—
Kapasia, Gazipur, Bangladesh	3	4.0	0.31	3.6	0.25	—	—	—	—	—
Rangpur, Bangladesh	3	4.2	0.32	4.0	0.29	—	—	—	—	—
Mithapukur, Bangladesh	3	4.6*	0.38	4.2	0.20	—	—	—	—	—
Pirgachha, Bangladesh	3	4.9	0.21	4.4	0.31	—	—	—	—	—

^*^Indicates that the grain yield is significantly higher than the parental variety at level, *p* = 0.05.

The head to head trials were conducted using management practices being followed by farmers in their particular locations. Although, these crop management practices and sources of seeds may vary over locations, the same management practices and seed source were used for any one pair planted in a single head to head trial. Besides, field operations including sowing, transplanting and harvesting of both varieties of a pair were carried out at the same time. The yield performance data for each variety in a trial was determined from the mean value of three randomly selected patches, each with an area of 10 × 10 m^2^. The grain yield was adjusted to 14% moisture content.

The effect of Sub1 gene on grain yield was estimated by the method suggested by Sheng and Li^30^, and Waza and Jaiswal^31^.

$${\rm{Effect}}\,{\rm{of}}\,{\rm{Sub}}1\,{\rm{gene}}=\frac{\bar{I}-\bar{N}}{\bar{N}}\times 100$$

where, $\bar{I}$ and $\bar{N}$ are the mean yield of the Sub1 variety and its recurrent parent, respectively. The positive value of the effect (deviation) indicates yield gain or yield advantage, whereas negative values indicate the penalty or loss in yield. The statistical significance of the deviation was calculated following the paired t-test using SPSS (Statistical Package for Social Sciences, version 16.0) software, at 0.05 level of significance. Any significant deviation across these large set of trials could be attributed to Sub1 QTL, as all pairs are considered near isogenic with high levels of genetic similarity within each pair^9,32^.

## Results

### Comparison of grain yield of Swarna-Sub1 and Swarna

**Figure 1:**
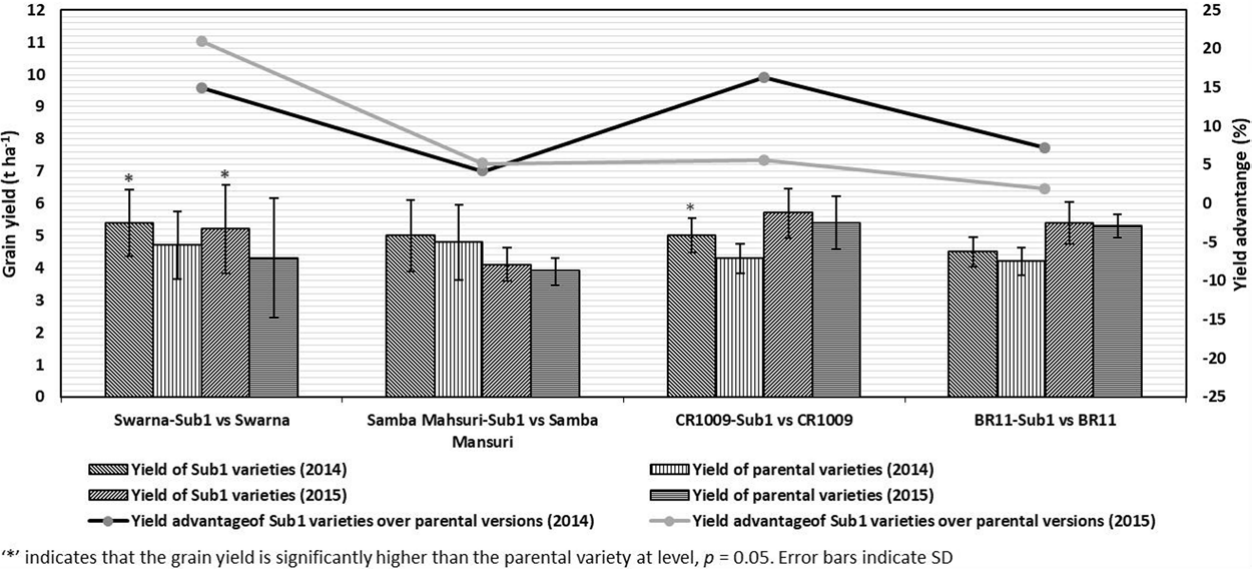
Comparison of mean grain yield (over locations) of Sub1 varieties and their recurrent parents.

Swarna-Sub1 produced significantly higher grain yield over Swarna in all the head to head trials during both years of the study, except at Kolkata, Puri, Torotpara and Rangpur during the wet season of 2014 and at Varanasi during the wet season of 2015 (Table 1). At these sites, Swarna was statistically alike to Swarna-Sub1 in grain yield during the respective years. The overall average yield of Swarna-Sub1 during 2014 was 5.4 t ha^−1^, which is higher than that of Swarna with the yield of 4.7 t ha^−1^ during the same year. Similarly, during 2015, the grain yields of Swarna-Sub1 and Swarna were, respectively, 5.2 t ha^−1^ and 4.3 t ha^−1^. The overall mean data over locations revealed a statistically significant yield advantage of 14.89% and 20.93% by Swarna-Sub1 over Swarna during 2014 and 2015, respectively (Fig. 1).

### Comparative yield of Samba Mahsuri-Sub1 and Samba Mahsuri

Samba Mahsuri-Sub1 had statistically higher grain yield over its non-Sub1 version at Puri and Hardinath during the wet seasons of 2014 and 2015, respectively. At other locations, statistically similar yields were recorded over both years of the study (Table 2). The average yield of Samba Mahsuri-Sub1 was 5.0 t ha^−1^ and 4.1 t ha^−1^ during the wet seasons of 2014 and 2015, respectively. However, for Samba Mahsuri, the average yield during these years was found to be 4.8 t ha^−1^ and 3.9 t ha^−1^, respectively. Although, overall mean yield (over locations) of Samba Mahsuri-Sub1 showed advantage of 4.17% and 5.13% over its non-Sub1 version during the wet seasons of 2014 and 2015, respectively, the values are not statistically significant (Fig. 1). Thus, Samba Mahsuri-Sub1 neither showed yield gain nor penalty over Samba Mahsuri under non-submergence conditions.

### Yield of CR1009-Sub1 and CR1009

Based on the head to head trials conducted during 2014, CR1009-Sub1 exhibited significantly higher grain yield over its non-Sub1 version at Annamalai. During this year, the trials were conducted at only one location using five farmers’ fields and average grain yield was 5.0 t ha^−1^ and 4.3 t ha^−1^ for CR1009-Sub1 and CR1009, respectively. In 2015, two locations were used with 10 farmers in one location and three in the second. The statistically similar yields of CR1009-Sub1 and its non-Sub1 version were obtained at both the locations during this year. The overall average yield of CR1009-Sub1 during 2015 was 5.7 t ha^−1^, which was statistically similar to CR1009, averaging 5.4 t ha^−1^. Though, the overall mean yield of CR1009-Sub1 showed advantage of 16.28% and 5.56% over its non-Sub1 version during 2014 and 2015, respectively, only the former value was statistically significant (Fig. 1).

### Yield Performance of BR11-Sub1 (BRRI Dhan-52) and BR11

The yield performance of BR11-Sub1 was statistically similar to its non-Sub1 version in all the head to head trials during both years of the study, except at Mithapukur during 2014 where BR11-Sub1 significantly out yielded BR11 (Table 4). The overall average yield of BR11-Sub1 was 4.5 t ha^−1^ and that of BR11 was 4.2 t ha^−1^ during the wet season of 2014. During 2015, the grain yield of 5.4 t ha^−1^ and 5.3 t ha^−1^ was obtained for BR11-Sub1 and BR11, respectively. BR11-Sub1 showed overall (over years) yield advantages of 7.14% and 1.89% over its non-Sub1 version during the wet seasons of 2014 and 2015, respectively (Fig. 1). However, both these values are statistically non-significant. Thus, BR11-Sub1 neither showed yield gain nor any yield penalty over its non-Sub1 version.

## Discussion

The farmers in submergence prone areas mainly cultivate improved rice varieties primarily due to their high yield potential and better grain quality. These varieties perform quite well even in the submergence prone areas during non-submergence years. However, during submergence these varieties lead to huge yield losses due to their inability to withstand the stress^33^. Recent innovations in agriculture have focused on making high yielding rice varieties more tolerant to submergence. Applying marker assisted breeding (MAB), the Sub1 (Submergence1) QTL has successfully been introgressed in several popular rice varieties grown in many parts of Indian subcontinent^23^. The incorporated gene (Sub1A) works conditionally (only in response to stress) and thus new Sub1 mega varieties have been anticipated to possess effective submergence tolerance without any undesirable effect on various traits of economic importance^25^.

The development of submergence tolerant versions of popular rice varieties allows communities to become more resilient to existing and growing flooding risks. This has made agricultural communities more resilient to flooding by partially stabilizing their production during flooding years^23^. Moreover, since the farmers are already familiar and well adapted with the popular varieties, introduction of their Sub1 versions will not require to inform them about various features and cultivation practices (of Sub1 varieties) as is required during the introduction of completely new varieties. Farmers also feel very commodious and show more enthusiasm in adopting the improved versions of their popular varieties. Furthermore, the Sub1 varieties reduce the risk of severe crop losses, thereby inducing the farmers to invest more on fertilizers and other agricultural inputs, and thus enhancing their seasonal earnings^34^. This also improves the propensity of farmers to experiment with latest technologies, including new varieties. These effects lead to the overall increase in farm output, regardless of the severity of the flood shock^24^. There are a number of additional impacts of Sub1 varieties that can be assessed in qualitative studies but have no quantitative evidence. This implies the impact on behavioral responses toward risk management, investment practices, household practices such as precautionary savings, etc. In addition, most of the farmers residing in highly submergence prone areas belong to socially and economically under privileged part of society. The introduction of Sub1 varieties in these areas has the potential to ensure food security and alleviate poverty from the socially disadvantaged communities^11^.

The Sub1 versions of popular rice varieties can withstand complete submergence for over two weeks and recover as water recedes^12^. Swarna-Sub1 is a Sub1 introgressed version of Swarna, a mega variety largely grown in Eastern India. This variety has been rigorously tested in farmer’s fields after being released in 2009^33^. Similarly, Samba Mahsuri-Sub1, CR1009-Sub1 and BR11-Sub1 are Sub1 introgressed versions of popular rice varieties grown over large areas in India, Nepal and Bangladesh^24^. These varieties have been tested and have huge potential under submergence conditions. However, the better performance of Sub1 varieties during submergence should not be at the cost of yield loss or penalty during non-flooding conditions as occurrence of flooding is not a regular phenomenon. The present study pertains to evaluate the effect of Sub1 QTL on grain yield of rice under non-submergence conditions, over years and locations.

Grain yield of Sub1 rice varieties under non-flooded conditions is expected to be equivalent to the respective recurrent parents. Any significant deviation of Sub1 varieties from respective parents in farmers’ fields could largely be attributed to the introgressed Sub1 QTLs as these pairs are near isogenic^9,32^. The data generated from different head to head trials revealed that some varieties, like Swarna-Sub1 have significantly higher yield than the original variety Swarna during both years of the study (Fig. 1). Similarly, the rice variety CR1009-Sub1 showed significant yield advantage over its recurrent parent during the wet season of 2014. The other two Sub1 varieties (Samba-Sub1 and BR11-Sub1) showed a marginal but insignificant enhancement in the grain yield. This deviation may be due to the manifold effects of the Sub1 locus expressed in a specific genetic background^35,36^. Introgression of other genes influencing grain yield along with Sub1 due to linkage or other small segments from the donor may also be responsible for the yield increment^37^. Furthermore, the Sub1 varieties being newly released have less chances of deteriorated seed and thereby leading to significantly higher grain yields over their respective non-Sub1 versions. In that case, seed quality ensures the yield advantage^38^. Moreover, the Sub1 QTL although being effective over environments and genetic backgrounds^1^, the Sub1 varieties should be evaluated for suitability before their large-scale dissemination in different geographical areas^39^.

The present study involves the evaluation and demonstration of submergence tolerant rice varieties in the form of head to head trials in contrast to the traditional methods used for the same purpose. The commonly used traditional method is to setup large scale cluster demonstrations where designated farmers cultivate the new variety and other farmers in the community are invited to visit and witness the outcome. Demonstration plots typically exhibit a treatment, but not a relevant counterfactual. As a consequence, they do not help farmers learn-by-comparing the new variety against their current technology. The farmers can witness the outcome, but cannot assess gain. Assessing gain would require setting up a control plot cultivated with the next best alternative available to the farmer under the same farmer-determined cultivation practices. Moreover, demonstration plots do not achieve relevance for social learning in not documenting the specific conditions under which the yield has been achieved. So, the visiting farmers are unable to compare these conditions to their own circumstances. In order to overcome these limitations, we have evaluated and demonstrated the flooding tolerant rice varieties in head to head trials where farmers can compare the Sub1 varieties with the respective non-Sub1 versions, under their own management. This also improve the farmers’ learning in their self-managed plots, thereby boosting their confidence. This helps to accelerate the adoption and acceptability of newly introduced varieties. Moreover, the relative superiority of submergence tolerant varieties in farmer’s field may enhance their dissemination, because most the farmers obtain seed from neighbors and relatives, and improved varieties spread rapidly through exchange once farmers are convinced for their superiority. Previous research that induced farmers to perform experiments on their own plots while keeping a comparison plot include Duflo *et al*.^40^. They made the farmers to apply two fertilizer recommendations on two plots while keeping a third plot cultivated without fertilizer. The researchers compensated the farmers for extra costs and followed them throughout the season. In our experiment, farmers were more than implementers in the sense that we let them to decide about the management practices for both counterfactual and the new technology.

The average grain yield of Sub1 varieties was either statistically similar or higher than their non-Sub1 counterparts. The submergence tolerant varieties in both the cases were promising as there was no case of overall yield penalty. Thus, the important characteristic of Sub1 varieties is their visible impact under submergence and no yield penalty under normal conditions over their non-Sub1 counterfactuals. There is a significant demand for a clearly superior technology that improves yield during stress years without imposing yield penalties during normal years. This justifies the need of replacing the popular rice varieties with their Sub1 introgressed versions, especially in the areas highly prone to submergence^1^.

Although, the Sub1 gene is already available in some popular rice varieties (Swarna, IR64, Samba Mahsuri, BR11 and CR1009), the need is to transfer this gene into other varieties that are popular in rainfed lowlands of the country^41^. In addition to Sub1, there are other minor QTLs that contribute significantly to submergence tolerance^27^. Some farmer’s varieties like FR13A and Khoda are more tolerant than Swarna-Sub1, and show rapid recovery after submergence^42^. In addition, some of the improved breeding lines like IR70213-10 can tolerate moderate water stagnation in addition to submergence. Introgression of submergence tolerance traits from local landraces and/or breeding lines into popular rice varieties would add value to these varieties and will help in obtaining sustainable yields in submergence prone areas^41^. There is a need for forward breeding approach to incorporate additional genes for submergence tolerance, and other genes for abiotic stresses that co-exisit in rainfed lowland areas, along with resistance to biotic factors.

## Conclusion

The average grain yield of Sub1 varieties was either statistically similar or higher than their non-Sub1 counterparts. The submergence tolerant varieties in all the cases were promising as there was no instance of overall yield penalty of the introgressed gene under non-flooding conditions. This is important for the farmers to accept a tolerant version of their old varieties. The development, dissemination and adoption of submergence tolerant version of popular rice varieties allow the communities to become more resilient to existing and growing flooding risks. Thus, there is a need to replace the popular rice varieties with their Sub1 versions especially in the areas highly prone to submergence. Moreover, the evaluation, demonstration and dissemination of the flooding tolerant rice varieties under head to head trials allow the farmers to compare the Sub1 varieties with normal ones, under their own management. These trials should be carried out at larger scale not only to assess genetic gains but also to create awareness among farmers.

## Electronic supplementary material

Dataset 1
