# Supplementary material for: No yield penalty under favorable conditions paving the way for successful adoption of flood tolerant rice
Source: Sci Rep. 2018 Jun 18;8:9245. doi: 10.1038/s41598-018-27648-y (PMC6006260; doi:10.1038/s41598-018-27648-y)
Supplement: Supplementary file 1 — Dataset 1 [file 41598_2018_27648_MOESM1_ESM.pdf]

Title:

No yield penalty under favorable conditions paving the way for successful adoption of flood tolerant rice

Authors:

Manzoor H. Dar[1,*], Najam W. Zaidi[1], Showkat A. Waza[2], Satish B. Verulkar[3], T. Ahmed[4], P. K. Singh[5], S. K. Bardhan Roy[6], Bedanand Chaudhary[7], Rambaran Yadav[8], Mirza Mofazzal Islam[9], Khandakar M. Iftekharuddaula[10], J. K. Roy[11], R. M. Kathiresan[12], B. N. Singh[13], Uma S. Singh[1] and Abdelbagi M. Ismail[14]

Affiliations:

[1]International Rice Research Institute (IRRI-India), NASC Complex, New Delhi, India

[2]Sher-e-Kashmir University of Agricultural Sciences & Technology of Kashmir (MCRS Sagam/Khudwani), J & K, India

[3]Indira Gandhi Krishi Vishwavidyalaya, Raipur, Chhattisgarh, India

[4]Assam Agricultural University, Jorhat, Assam, India

[5]Banaras Hindu University, Varanasi, Uttar Pradesh, India

[6]Center for Strategic Studies, Kolkata, West Bengal, India

[7]Regional Agricultural Research Station, NARC, Tarahara, Nepal

[8]National Rice Research Program, Hardinath, Nepal

[9]Bangladesh Institute of Nuclear Agriculture, Mymensingh, Bangladesh

[10]Bangladesh Rice Research Institute, Gazipur, Bangladesh

[11]Association for Integrated Development (AID), Bhubaneswar, Odisha, India

[12]Annamalai University, Annamalainagar, India

[13]Centre for Research and Development (CRD), Gorakhpur, UP, India

[14]International Rice Research Institute (IRRI), Los Banos, Philippines

[*]Corresponding author: m.dar@irri.org

Supplementary Tables

Table I. Yield advantage of Swarna-Sub1 over Swarna under head to head trials during the wet season of 2014.

Locations	No. of head to head trials	Yield advantage (%) under head to head trials							
		1	2	3	4	5	6	Mean	SD
Kolkata, West Bengal, India	3	4.23	0.00	4.48	-	-	-	2.91	2.52
Gorakhpur, Uttar Pradesh, India	3	50.00	21.95	6.82	-	-	-	24.79	21.91
Titabar, Assam, India	5	15.38	3.92	3.85	9.43	16.33	-	9.73	6.00
Dhamtari, Chhattisgarh, India	3	18.18	9.62	26.00	-	-	-	17.83	8.20
Puri, Nimapara, Odisha, India	6	10.42	9.62	13.73	13.21	6.38	5.88	9.93	3.30
Tarhara, Sunsari, Nepal	3	81.08	90.91	93.55	-	-	-	88.12	6.57
Hardinath, Nepal	3	27.50	13.16	21.62	-	-	-	20.87	7.21
Torotpara, Gazipur, Bangladesh	3	0.00	-18.60	2.56	-	-	-	-5.60	11.55
Rangpur, Bangladesh	3	9.30	2.38	0.00	-	-	-	4.03	4.83

Table II. Yield advantage of Swarna-Sub1 over Swarna under head to head trials during the wet season of 2015.

Locations	No. of head to head trials	Yield advantage (%) under head to head trials						
		1	2	3	4	5	Mean	SD
Varanasi, Uttar Pradesh, India	3	7.35	1.43	1.59	-	-	3.48	3.38
Tarhara, Sunsari, Nepal	5	21.62	17.14	54.84	33.33	40.00	32.74	15.05
Hardinath, Nepal	3	28.21	26.47	30.56	-	-	28.44	2.05

Table III. Yield advantage of Samba Mahsuri-Sub1 over Samba Mahsuri under head to head trials during the wet season of 2014.

Locations	No. of head to head trials	Yield advantage (%) under head to head trials				
		1	2	3	Mean	SD
Kolkata, West Bengal, India	3	3.57	3.28	3.33	3.39	0.16
Gorakhpur, Uttar Pradesh, India	3	-30.36	0.00	-4.55	-13.29	16.37
Puri, Odisha, India	3	6.45	6.90	6.78	6.70	0.23
Tarhara, Sunsari, Nepal	3	19.35	22.22	33.33	24.74	7.38
Hardinath, Nepal	3	13.95	9.52	2.50	8.80	5.78

Table IV. Yield advantage of Samba Mahsuri-Sub1 over Samba Mahsuri under head to head trials during the wet season of 2015.

Locations	No. of head to head trials	Yield advantage (%) under head to head trials											
		1	2	3	4	5	6	7	8	9	10	Mean	SD
Kolkata, West Bengal, India	3	0.00	9.76	4.88	-	-	-	-	-	-	-	5.00	4.88
Puri, Odisha, India	5	2.63	0.00	-5.71	2.86	0.00	-	-	-	-	-	0.00	3.45
Varanasi, Uttar Pradesh, India	3	7.84	4.35	6.98	-	-	-	-	-	-	-	6.43	1.82
Annamalai, Tamil Nadu, India	10	0.00	5.88	0.00	-2.33	4.55	0.00	4.65	4.88	2.78	5.88	2.60	2.97
Tarhara, Sunsari, Nepal	5	6.25	2.78	5.13	8.11	6.06	-	-	-	-	-	5.65	1.94
Hardinath, Nepal	3	17.07	16.67	18.42	-	-	-	-	-	-	-	17.39	0.92

Table V. Yield advantage of CR1009-Sub1 over CR1009 under head to head trials during the wet season of 2014.

Locations	No. of head to head trials	Yield advantage (%) under head to head trials						
		1	2	3	4	5	Mean	SD
Annamalai, Tamil Nadu, India	5	9.76	19.05	23.08	17.39	8.51	15.35	6.23

Table VI. Yield advantage of CR1009-Sub1 over CR1009 under head to head trials during the wet season of 2015.

Locations	No. of head to head trials	Yield advantage (%) under head to head trials											
		1	2	3	4	5	6	7	8	9	10	Mean	SD
Kolkata, West Bengal, India	3	4.76	6.25	9.09	-	-	-	-	-	-	-	6.72	2.20
Annamalai, Tamil Nadu, India	10	1.96	6.00	5.45	1.64	8.33	3.39	1.92	3.51	5.08	5.17	4.27	2.15

Table VII. Yield advantage of BR11-Sub1 over BR11 under head to head trials during the wet season of 2014.

Locations	No. of head to head trials	Yield advantage (%) under head to head trials				
		1	2	3	Mean	SD
Torotpara, Gazipur, Bangladesh	3	-11.36	5.00	2.56	-1.63	8.83
Valuka, Gazipur, Bangladesh	3	4.00	2.17	3.92	3.40	1.03
Kapasia, Gazipur, Bangladesh	3	10.26	14.71	2.78	9.17	6.03
Rangpur, Bangladesh	3	7.89	6.98	5.26	6.72	1.34
Mithapukur, Bangladesh	3	16.67	5.00	9.09	10.32	5.92
Pirgachha, Bangladesh	3	14.63	8.51	11.63	11.45	3.06

Table VIII. Yield advantage of BR11-Sub1 over BR11 under head to head trials during the wet season of 2015.

Locations	No. of head to head trials	Yield advantage (%) under head to head trials									
		1	2	3	4	5	6	7	8	Mean	SD
Titabar, Assam, India	8	8.00	3.92	7.14	7.41	3.39	3.57	1.79	1.75	4.56	2.53
Torotpara, Gazipur, Bangladesh	3	2.00	4.35	-13.21	-	-	-	-	-	-	9.53
